# Use of Droplet Digital PCR for Estimation of Fish Abundance and Biomass in Environmental DNA Surveys

**DOI:** 10.1371/journal.pone.0122763

**Published:** 2015-03-23

**Authors:** Hideyuki Doi, Kimiko Uchii, Teruhiko Takahara, Saeko Matsuhashi, Hiroki Yamanaka, Toshifumi Minamoto

**Affiliations:** 1 Institute for Sustainable Sciences and Development, Hiroshima University, Higashi-Hiroshima, Japan; 2 Faculty of Pharmacy, Osaka Ohtani University, Tondabayashi, Japan; 3 Graduate School of Integrated Arts and Sciences, Hiroshima University, Higashi-Hiroshima, Japan; 4 Department of Environmental Solution Technology, Faculty of Science and Technology, Ryukoku University, Otsu, Japan; 5 Graduate School of Human Development and Environment, Kobe University, Kobe, Japan; Central Michigan University, UNITED STATES

## Abstract

An environmental DNA (eDNA) analysis method has been recently developed to estimate the distribution of aquatic animals by quantifying the number of target DNA copies with quantitative real-time PCR (qPCR). A new quantitative PCR technology, droplet digital PCR (ddPCR), partitions PCR reactions into thousands of droplets and detects the amplification in each droplet, thereby allowing direct quantification of target DNA. We evaluated the quantification accuracy of qPCR and ddPCR to estimate species abundance and biomass by using eDNA in mesocosm experiments involving different numbers of common carp. We found that ddPCR quantified the concentration of carp eDNA along with carp abundance and biomass more accurately than qPCR, especially at low eDNA concentrations. In addition, errors in the analysis were smaller in ddPCR than in qPCR. Thus, ddPCR is better suited to measure eDNA concentration in water, and it provides more accurate results for the abundance and biomass of the target species than qPCR. We also found that the relationship between carp abundance and eDNA concentration was stronger than that between biomass and eDNA by using both ddPCR and qPCR; this suggests that abundance can be better estimated by the analysis of eDNA for species with fewer variations in body mass.

## Introduction

Species distribution and biomass are fundamental for the evaluation of population dynamics and community structure in an ecosystem. However, surveying species distribution is laborious, especially in animal species that are difficult to catch, such as fish in aquatic ecosystems. To address this issue, environmental DNA (eDNA) analysis methods have been recently developed to estimate the distribution of aquatic animals [1–12]. The application of these methods has been expanded to many types of animals, including amphibians [1, 2], fish [3–10], mollusks [11], insects [12,13], and crustaceans [14], in various ecosystems such as lakes/ponds [3, 5, 6, 9, 10, 14], rivers [2, 4, 13, 15], and oceans [7, 8]. These studies used PCR techniques for the detection of eDNA of the target species, thereby assessing the presence/absence of the species.

A few researchers have tried to estimate species biomass and abundance on the basis of the eDNA concentration in the water by using quantitative real-time PCR (qPCR) [5, 12, 16]. They found a positive correlation between the abundance or biomass and the eDNA concentration. Thus, the eDNA analysis method can be applied to evaluate the abundance/biomass of the species in aquatic habitats. However, no quantitative eDNA technique has been fully established to estimate the abundance/biomass of the target species. Many researchers are uncertain how long eDNA would persist in the wild because of a number of environmental factors [5, 10, 12, 16, 17]. In addition, there are methodological limitations to quantifying eDNA concentration, which have not been thoroughly addressed.

Since qPCR uses an indirect measurement that refers to the cycle threshold (Ct), the method has potential limitations with respect to data accuracy and reproducibility [18, 19]. Recently, a new PCR method that directly quantifies DNA copies has been developed [20]; droplet digital PCR (ddPCR), also known as a “third-generation” PCR, is an emerging DNA detection method that provides absolute quantification of target DNA without a standard curve of the reference. DNA is partitioned into approximately 20,000 droplets, some of which ideally contain one or few copies of the target DNA. The PCR occurs in each droplet, and end-point PCR amplification is detected using fluorescence probes [20]. ddPCR can detect differences as low as 1.25-fold, which is more accurate than the 2-fold differences detected using qPCR [19, 20]. To date, ddPCR has been used instead of qPCR [21] to obtain absolute quantification of a virus [22, 23], bacteria [19, 24], and animal cells [19, 21]. Recently, ddPCR was used to quantify the eDNA concentration of fish [25], and smaller variations in ddPCR estimates than in qPCR ones were reported. Thus, ddPCR would more accurately quantify eDNA concentration, enhancing the estimation capability of species abundance/biomass in eDNA surveys.

In this study, we compared the methodological advantages of ddPCR over those of conventional qPCR for eDNA quantification. We specifically focused on the quantification accuracy of low eDNA concentrations because eDNA concentrations of the target species recovered from field samples are often very low. We conducted mesocosm experiments by using the common carp, *Cyprinus carpio* L.; we used different numbers of carp and measured eDNA concentration in the water by using qPCR and ddPCR. We compared the quantification accuracy of both methods to estimate fish abundance and biomass on the basis of eDNA.

## Materials and Methods

### Mesocosm experiment

We purchased common carp, *Cyprinus carpio* L., from a fish farm and used them for performing mesocosm experiments with twelve concrete outdoor tanks (180 × 100 × 33 cm, volume: 450 L). Each mesocosm was filled with tap water one week before the experiment. At day 0 of the experiment (September 20, 2013), we introduced different numbers of carp into the mesocosms: 0 (as a negative control), 3, 4, 5, 7, 9, 11, 17, 25, 38, 56, and 85 (in total, 260 individuals). Before introducing the carp (day 0), we measured water quality (at a depth of 5 cm), water temperature, dissolved oxygen (DO), pH, and electric conductivity (EC). Water temperature and DO were measured with an optimal DO sensor (ProODO; YSI, Yellow Spring, OH, USA). The pH and EC were measured with Twin pH B-212 and Twin Conductivity B-171, respectively (Horiba, Ltd., Kyoto, Japan). Water temperature ranged from 24.8 to 25.3°C (25.1 ± 0.14°C, mean ± 1 SD). DO, pH, and EC ranged from 8.36 to 9.49 mg L^-1^ (8.93 ± 0.35), 8.6 to 9.2 (9.06 ± 0.21), and 1.30 to 1.49 μS m^-1^ (1.40 ± 0.049), respectively. Thus, we confirmed that water quality was similar in the mesocosms. We measured the wet weight of each carp at the end of the experiment (27.6 ± 8.2 g, mean ± 1 SD) and calculated the biomass in each mesocosm. The dead individuals were removed immediately from the mesocosm and frozen. In total, 11 individuals died in the mesocosms with higher number of individuals in the first three days of the experiment, and they were removed immediately. For the statistical analysis, we used carp abundance and biomass, which were measured at the end of the experiment. All experimental procedures were conducted according to the current laws of Japan and approved by the Animal Research Committee of Hiroshima University, and we followed the guidelines for experimental vertebrate animals (Hiroshima University, Law No. 102, 1 Mar. 2012).

### eDNA sampling

On days 1, 2, and 3 of the experiment, between 11:00 and 14:00, we collected 15 mL of water from each mesocosm in a 50-mL plastic centrifuge tube with a plastic pipette. The water was sampled from the center of the mesocosm at a depth of 5 cm. Immediately after sampling, 1.5 mL of 3 mol L^-1^ sodium acetate (pH 5.2) and 33 mL of absolute ethanol were added to each water sample, and the tubes were stored at—20°C until DNA extraction. We used 15 mL of deionized water as a blank every sampling day to verify the absence of cross contamination during the experimental procedures, i.e., water sampling, eDNA extraction, and qPCR/ddPCR. DNA extraction methods were adapted from Ficetola et al. (2008) [1]. The tube was centrifuged at 10,000 × *g* for 1 h at 4°C to recover DNA fragments and cellular and suspended materials. eDNA from the resulting pellet was extracted using the DNeasy Blood & Tissue Kit (Qiagen, Hilden, Germany), according to the manufacturer’s instructions, and the DNA was finally eluted with 100 μL of Buffer AE. The DNA solution was stored in a 1.5-mL microtube at −25°C until PCR analysis. DNA extractions and PCR were performed in different rooms.

### qPCR

qPCR for mitochondrial cytochrome *b* gene fragments was performed according to the method described by Takahara et al. [5]. Each PCR reaction mixture (20 μL) contained 2 μL of DNA solution with 900 nM forward and reverse primers (F, 5′-GGTGGGTTCTCAGTAGACAATGC-3′; R, 5′-GGCGGCAATAACAAATGGTAGT-3′) and 125 nM TaqMan probe (5′-FAM-CACTAACACGATTCTTCGCATTCCACTTCC-TAMRA-3′) in a 1× PCR master mix (TaqMan Gene Expression Master Mix, Life Technologies, Foster City, CA, USA). PCR reactions were performed in triplicate under thermal cycler conditions of 2 min at 50°C, 10 min at 95°C, and 55 cycles of 15 s at 95°C and 60 s at 60°C in a StepOnePlus Real Time PCR system (Life Technologies). Three wells were used as a no-template negative control for all qPCR assays; an amplification signal was not observed in these wells and the blank samples. The specificity of probe and primers was previously confirmed by Takahara et al. [5], where the qPCR amplicons in all positive samples were directly sequenced and confirmed as being from common carp.

The PCR products of the target sequences were cloned into the pGEM-T Easy Vector (Promega, Tokyo, Japan). The vector was digested with a restriction enzyme (*Eco*RI) to make it linear, and it was then used as a standard for quantification. A dilution series of the standard, 6 × 10^1^ to 6 × 10^4^ copies per 20 μL PCR reaction mixture, were amplified in triplicate in all qPCR assays to produce standard curves for quantification. The R^2^ values of the standard curve for the qPCR experiments ranged from 0.982 to 0.993. The mean and coefficients of variation (CV, %) values were calculated using the triplicate quantitative values for each sample and then used for statistical analyses.

### ddPCR

We performed ddPCR by using the same primers and probe as those used for qPCR. Each ddPCR reaction mixture (20 μL) contained 2 μL of DNA template, 900 nM of each primer, and 125 nM TaqMan probe in a 1× Bio-Rad Supermix (Bio-Rad, Hercules, CA, USA), which was mixed with Bio-Rad droplet generator oil and partitioned into 15,000–20,000 droplets by using the Bio-Rad QX-100 droplet generator (Bio-Rad). The droplets of individual samples were separately applied to each well of a 96-well PCR reaction plate. PCR was performed in the 96-well plate sealed with pierceable sealing foil by using the GeneAmp 9700 thermocycler (Life Technologies). PCR conditions were 10 min at 95°C, 40 cycles of denaturation for 30 s at 95°C and extension for 60 s at 60°C with ramp rate of 2.5°C s^-1^, followed by 10 min at 95°C and a hold at 4°C. After PCR amplification, the plate was transferred to the Bio-Rad QX-100 droplet reader (Bio-Rad). Each droplet in the well was checked for TaqMan fluorescence to count the number of droplets that yielded positive/negative results.

We used Bio-Rad’s QuantaSoft software version 1.3.2.0 to quantify the copies of target DNA in μL^-1^. Threshold for a positive signal was determined according to the QuantaSoft instructions. Any droplet beyond the fluorescence threshold was counted as a positive event. Blank samples showed negative results for DNA copies. All samples were run in triplicates, and we used the mean and CV (%) values for statistical analyses.

### Statistical analyses

We evaluated the correlation between eDNA concentration and abundance/biomass of carp by using a Type II regression with a standardized major axis method. Type II regression can treat two variables (x and y) with equal magnitude of random variation. We evaluated the regression for all data collected on the three experimental days, because the trends were very similar between the days and the slopes were not significantly different from each other in our preliminary analysis (p > 0.05). eDNA concentration, carp abundance and biomass were log_10_-transformed to normalize the values on the basis of the results of the Shapiro-Wilk normality test (α = 0.05). The R^2^ values of Type II regressions for qPCR and ddPCR were statistically compared using Fisher’s z-test. To compare the mean CVs of qPCR and ddPCR, we used Welch’s t-test. The slopes and intercepts of the Type II regressions between CVs and eDNA concentrations were compared using the 95% confidence intervals between qPCR and ddPCR (α = 0.05). All statistical analyses were performed at a significance level of α = 0.05. All statistical analyses and graphics were conducted using R ver. 3.1.0 [26].

## Results

### eDNA quantification by using qPCR and ddPCR

**Fig 1:**
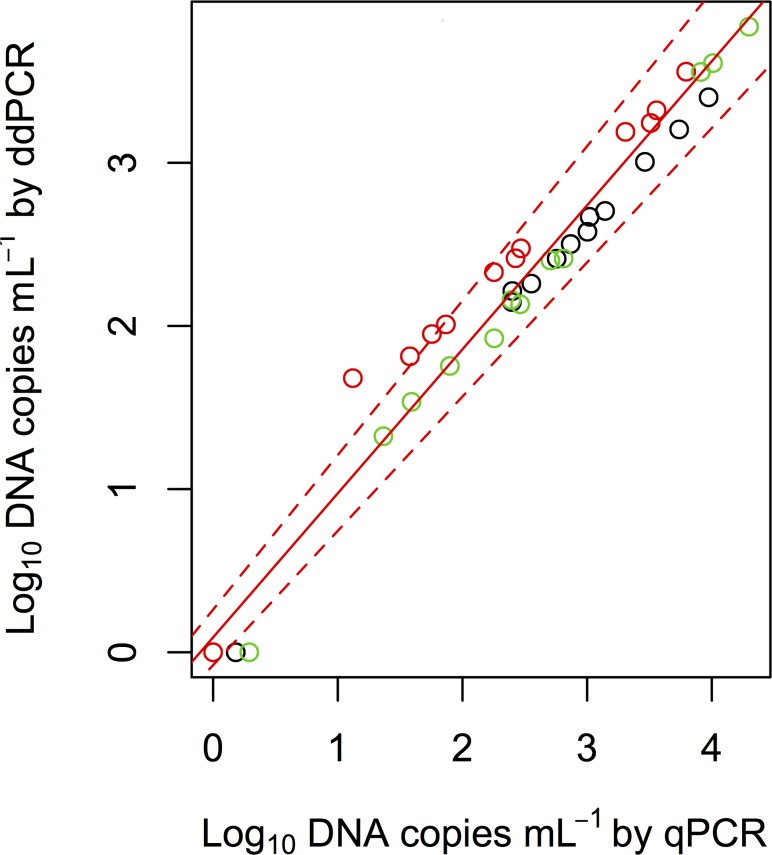
Relationships between eDNA concentrations of common carp in mesocosms that were measured using qPCR and ddPCR. Black, red, and green symbols indicate samples collected on days 1, 2, and 3, respectively. Solid red and dashed red lines indicate Type II regression and 95% CI, respectively.

**Fig 2:**
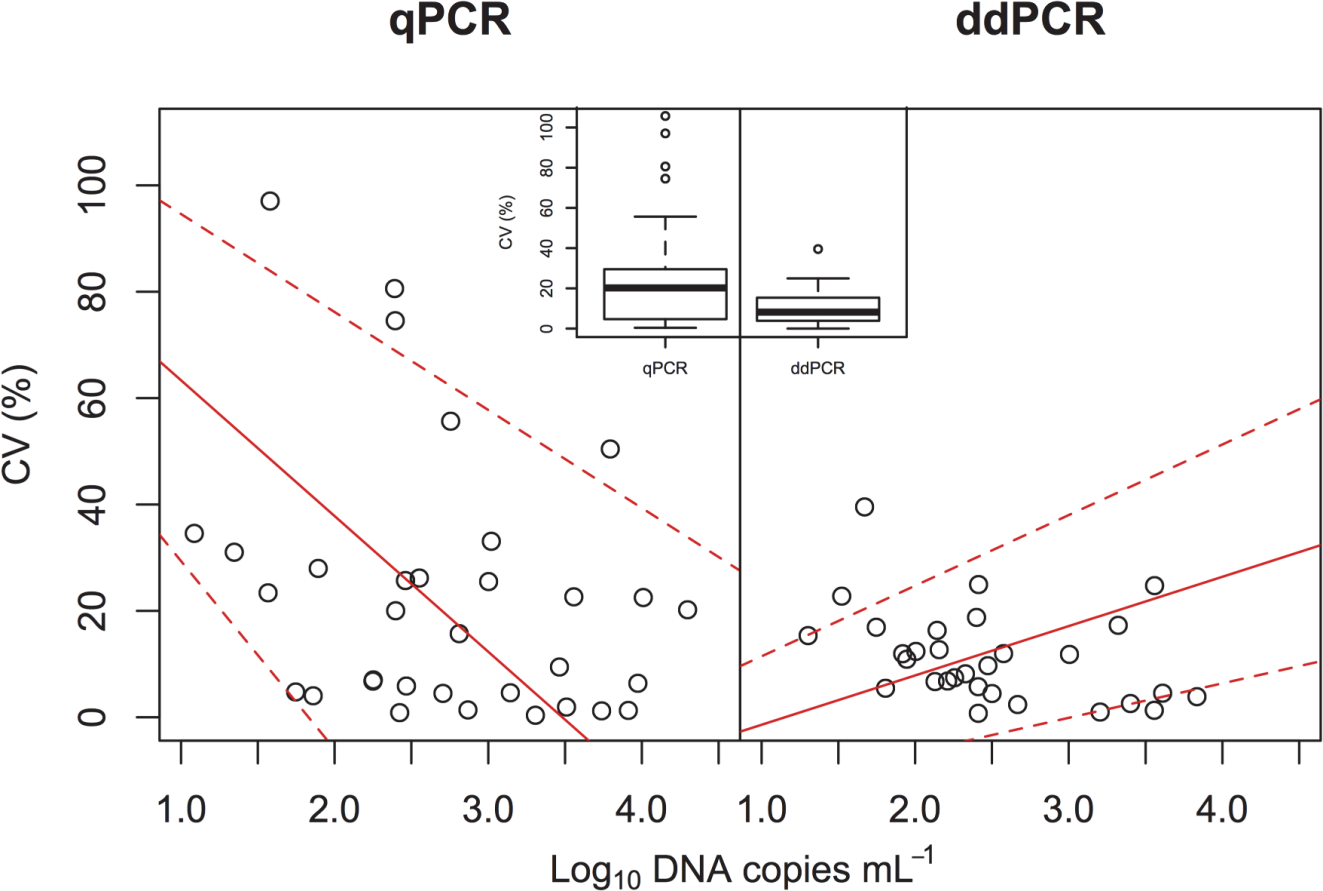
Relationships between eDNA concentrations of common carp and coefficients of variation (CV, %) measured using qPCR and ddPCR. Solid red and dashed red lines indicate Type II regression and 95% CI, respectively. Boxes in the box plot indicate median ± quartiles, and points indicate the outliers. The means of CVs were significantly different by Welch’s t-test (t = –2.98, p = 0.0047).

We compared the eDNA concentrations of common carp in the mesocosms that were measured using qPCR and ddPCR (Fig. 1), and detected a significant positive correlation between qPCR and ddPCR methods (Type II regression, R^2^ = 0.961, p < 0.0001). Welch’s t-test (t = –2.98, p = 0.005) showed that the coefficients of variations in the eDNA concentrations measured using ddPCR (10.3 ± 8.9%, mean ± 1 SD) were significantly smaller than those measured using qPCR (25.0 ± 27.6%, Fig. 2). The slope of Type II regression in qPCR (y = –25.5*x* + 88.9) was significantly lower than that in ddPCR (y = 9.27*x* – 10.7) (p < 0.05); the intercept in qPCR was significantly higher than that in ddPCR (p < 0.05), indicating that the CVs of qPCR were higher at lower eDNA concentrations than ddPCR. In fact, higher CV values, >40%, were found only in qPCR, especially at lower eDNA concentrations (Fig. 2).

### Abundance/biomass estimation by using qPCR and ddPCR

**Fig 3:**
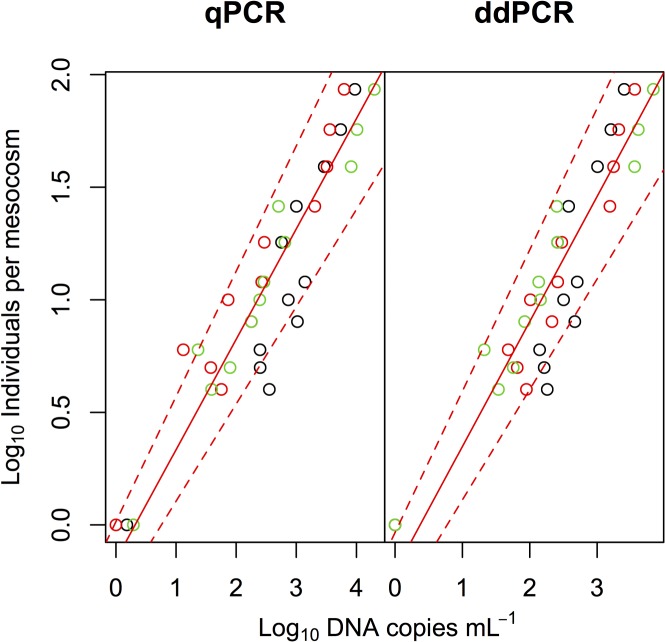
Relationships between eDNA concentrations of common carp and their abundance in the mesocosm experiment. Black, red, and green symbols indicate samples collected on days 1, 2, and 3, respectively. Solid red and dashed red lines indicate Type II regression and 95% CI, respectively.

We evaluated the relationship between the eDNA concentrations of common carp and carp abundance in the mesocosms (Fig. 3, S1 Table). We detected a significantly positive correlation between carp abundance and DNA copies estimated using qPCR (Type II regression, R^2^ = 0.868, p < 0.0001, y = 0.490*x –* 0.156). The correlation between carp abundance and DNA copies estimated using ddPCR was significantly positive with a larger R^2^ value than qPCR (R^2^ = 0.874, p < 0.0001, y = 0.555*x* – 0.207), but the difference in R^2^ values for qPCR and ddPCR was not statistically significant (Fisher’s z test, z = 0.12, p = 0.091).

**Fig 4:**
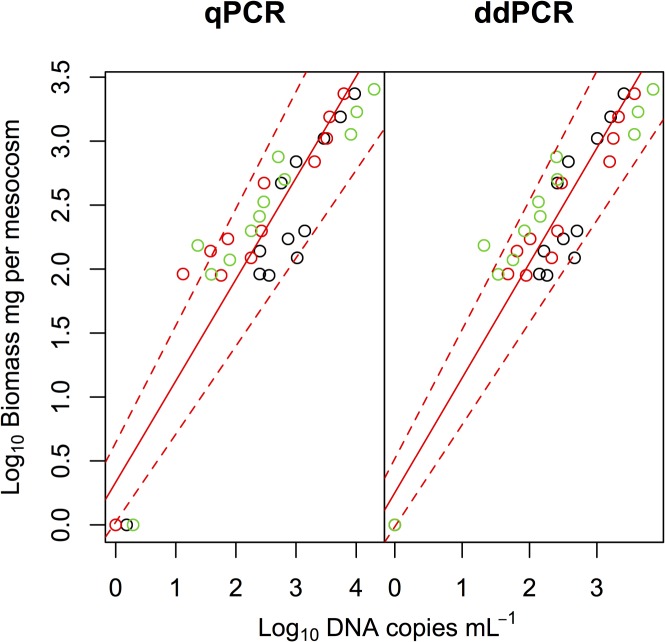
Relationships between eDNA concentrations of common carp and their biomass in the mesocosm experiment. Black, red, and green symbols indicate samples collected on days 1, 2, and 3, respectively. Solid red and dashed red lines indicate Type II regression and 95% CI, respectively.

We also compared the eDNA concentrations and biomass (Fig. 4) and found a significant positive correlation for both qPCR (Type II regression, R^2^ = 0.783, p < 0.0001, y = 0.792*x* + 0.333) and ddPCR (R^2^ = 0.886, p < 0.0001, y = 0.898*x* + 0.251). The R^2^ value was significantly larger for ddPCR than for qPCR (Fisher’s z test, z = 2.0, p = 0.040).

## Discussion

We found that both qPCR and ddPCR were able to quantify the concentration of carp eDNA along with abundance and biomass gradients (Figs. 3 and 4). However, similar to previous studies [18–20, 22], we found lower variation in ddPCR measurements than in qPCR measurements (Fig. 2). The CVs in qPCR were sometimes larger than 50% when estimating eDNA concentration, whereas the CVs in ddPCR were about 10% because ddPCR is able to detect differences as low as 1.25-fold [19]. Thus, ddPCR would be more efficient than qPCR in accurately estimating fish abundance/biomass by using eDNA.

A previous study on eDNA [25] found that both qPCR and ddPCR yielded similar results for the quantification of eDNA of goby fish and its abundance (3, 12, and 33 individuals in 1500-L mesocosms); these findings were consistent with our results, even though we used a different eDNA extraction method and target species. We further assessed the quantification accuracy of qPCR and ddPCR at low eDNA concentration by creating mesocosms with a wider range and a more gradual gradient in fish abundance. ddPCR would be more accurate in quantifying low quantities of eDNA than qPCR (Fig. 2). Similar results were reported when qPCR and ddPCR measurements for HIV DNA were compared [20]. Previous studies using ddPCR have also reported the high sensitivity of ddPCR in detecting lower quantities of DNA copies of bacteria [24] and cells [19]. In addition, ddPCR has an advantage in measuring a number of samples in multiple runs because it does not require DNA standard molecules which can be degraded and produce variations in results between runs, especially at low DNA concentration. Usually, eDNA samples from natural habitats have low eDNA concentrations of each species living in such habitats. Since ddPCR can quantify few DNA copies with high precision, it can predict species abundance and biomass in eDNA surveys better than qPCR.

The accuracy of the estimation of eDNA concentration in water is not only affected by the quantification method but also by other processes involved in the recovery of DNA from water, including water sampling, eDNA extraction, and DNA purification. In fact, our mesocosm experiment found stronger correlations between the eDNA concentration and biomass than our previous experiments with common carp that were conducted in aquariums and ponds [5], probably because of the differences in the eDNA extraction methods; ethanol precipitation was used in this study and ultra-centrifugation and membrane filtration were used in the previous study [5]. Ethanol precipitation is better than filtering methods for estimating eDNA concentration in water, because the filtering methods cannot be used to collect all eDNA fragments from the water sample [27]. However, because ethanol precipitation can process only a small amount of water, the method is suitable only when the eDNA concentration of the target species is high. We need to select appropriate methods for eDNA collection in order to increase the accuracy and sensitivity of eDNA quantification.

The inhibitory substances in natural samples had little effect on DNA quantification in ddPCR, because the end-point PCR amplification in each droplet can be detected independent of the amplification efficiency [28]. Thus, ddPCR is more suitable for the measurement of natural eDNA samples than qPCR. Our mesocosm system contained common carp alone in aged tap water. Therefore, the conditions were different from those in natural habitats where water usually contains more particulate and dissolved substances and DNA from other organisms. Although we did not test the advantage of ddPCR by using natural eDNA samples, our results encourage the use of ddPCR in the field. ddPCR can amplify and measure longer DNA fragments, i.e., ~450 bp [19], whereas qPCR can generally measure fragments <150 bp in length. When longer DNA fragments are used to identify a targeted species, researchers should consider using ddPCR.

The estimation accuracy of our regression model was higher for carp abundance than for biomass. Thomsen et al. (2012) also found a clear correlation between eDNA concentration and abundance in their experiment using organisms of similar body mass [12]. eDNA in the water is released from skin, feces, or scales. The amount of such materials released from the individuals is related to a greater extent to the number of individuals than to their total biomass if the individual body mass is similar within the population. In our mesocosm experiment, we used carp individuals of similar body mass. In natural habitats, however, the body mass of a species generally varies. Thus, eDNA estimation for organism abundance in the field is more complex. However, for species with small variations in body mass in a particular season, such as fish used for aquaculture and juvenile fish introduced by humans, eDNA can precisely estimate their abundance.

Recently, another new molecular technique, i.e., high-throughput sequencing, has been applied for eDNA studies [12, 29, 30]. DNA metabarcoding approach may have advantages in simultaneous detection of multiple species and cost effectiveness [29, 30]. In contrast, quantitative PCR techniques, such as real-time and digital PCR, would be more reliable to estimate biomass or abundance of specific species. By combining multiple technologies, we can develop better eDNA methods to evaluate the distribution and abundance/biomass of species and communities.

In conclusion, ddPCR would be superior to qPCR in quantifying eDNA concentration in water for the estimation of fish abundance and biomass because it provides a more accurate quantification than qPCR, especially at lower DNA concentrations. However, ddPCR is more expensive and time-consuming than qPCR. The price for our ddPCR method is about $14.1 per sample measured in triplicate (S2 Table), and 5 h 40 mins were required from eDNA extraction to completion of the analysis (S3 Table). In addition, the ddPCR apparatus is much more expensive than that of real-time PCR. Further development of ddPCR for practical use is required. ddPCR has other merits, such as fewer analysis errors and it is less affected by inhibitory substances. Thus, ddPCR may become the next standard method to quantify eDNA concentration in eDNA studies.

## Supporting Information

S1 ARRIVE checklist (DOC)

S1 MIQE checklist (XLS)

S1 Table All data which used in this study. Biomass and individuals per mesocosm indicate the final total biomass of the carp in each mesocosm and individuals, which introduced in the mesocosms, respectively.
(DOC)

S2 Table Costs (US$*) for ddPCR and qPCR measurements. (DOC)

S3 Table Estimated time for ddPCR and qPCR measurements. (DOC)
