# Supplementary material for: Use of Droplet Digital PCR for Estimation of Fish Abundance and Biomass in Environmental DNA Surveys
Source: PLoS One. 2015 Mar 23;10(3):e0122763. doi: 10.1371/journal.pone.0122763 (PMC4370432; doi:10.1371/journal.pone.0122763)
Supplement: S1 ARRIVE checklist — (DOC) [file pone.0122763.s001.doc]

**The ARRIVE Checklist**

**TITLE**

**1 Provide as accurate and concise a description of the content of the article as possible.**

Use of droplet digital PCR for estimation of fish abundance and biomass in environmental DNA surveys

**ABSTRACT**

**2 Provide an accurate summary of the background, research objectives (including details of the species or strain of animal used), key methods, principal findings, and conclusions of the study.**

An environmental DNA (eDNA) analysis method has recently been developed to estimate the distribution of aquatic animals by quantifying the number of target DNA copies with quantitative real-time PCR (qPCR). A new quantitative PCR technology, droplet digital PCR (ddPCR), partitions PCR reactions into thousands of droplets and detects the amplification in each droplet, thereby allowing direct quantification of target DNA. We evaluated the quantification accuracy of qPCR and ddPCR to estimate species abundance and biomass using eDNA in mesocosm experiments involving different numbers of common carp. We found that ddPCR quantified the concentration of carp eDNA along with carp abundance and biomass more accurately than qPCR, especially at low eDNA concentrations. In addition, the errors in the analysis were smaller in ddPCR than in qPCR. Thus, ddPCR is better suited to measure eDNA concentration in water, providing more accurate results on the abundance and biomass of target species than qPCR. We also found that the relationship between carp abundance and eDNA concentration was stronger than that between biomass and eDNA both in ddPCR and qPCR; this suggests that the abundance can be well estimated by the analysis of eDNA for species with fewer variations in body mass.

**INTRODUCTION**

**Background**

**3 a. Include sufficient scientific background (including relevant references to previous work) to understand the motivation and context for the study, and explain the experimental approach and rationale.**

**b. Explain how and why the animal species and model being used can address the scientific objectives and, where appropriate, the study’s relevance to human biology.**

In this study, we evaluated the methodological advantages of ddPCR in eDNA quantification in comparison with the conventional qPCR. We specifically focused on the quantification accuracy of low eDNA concentrations because the eDNA concentration of target species recovered from field samples is often very low. We conducted mesocosm experiments using common carp, *Cyprinus carpio* L., in which different numbers of carp were introduced and the eDNA concentration in the water was measured by qPCR and ddPCR. We compared the quantification accuracy of both methods to estimate fish abundance and biomass based on eDNA.

**Objectives**

**4 Clearly describe the primary and any secondary objectives of the study, or specific hypotheses being tested.**

- The ddPCR would more accurately quantify the eDNA concentration, enhancing the estimation capability of the species abundance/biomass in eDNA surveys.
- To compare ddPCR and qPCR methods, we conducted mesocosm experiments using common carp, *Cyprinus carpio* L., in which different numbers of carp were introduced and the eDNA concentration in the water was measured by qPCR and ddPCR.

**METHODS**

**Ethical statement**

**5 Indicate the nature of the ethical review permissions, relevant licenses (e.g. Animal [Scientific Procedures] Act 1986), and national or institutional guidelines for the care and use of animals, that cover the research.**

All experimental procedures were conducted according to the current laws of Japan and approved by the Animal Research Committee of Hiroshima University according to the guidelines of experimental vertebrate animals (Hiroshima University, Law No. 102, 1 Mar. 2012).

**Study design**

**6 For each experiment, give brief details of the study design, including:**

**a. The number of experimental and control groups.**

**b. Any steps taken to minimise the effects of subjective bias when allocating animals to treatment (e.g., randomisation procedure) and when assessing results (e.g., if done, describe who was blinded and when).**

**c. The experimental unit (e.g. a single animal, group, or cage of animals).**

**A time-line diagram or flow chart can be useful to illustrate how complex study designs were carried out.**

**a.** We used common carp, *Cyprinus carpio* L., which was purchased from a fish farm, in mesocosm experiments in twelve concrete outdoor tanks (180 × 100 × 33 cm, volume: 450 L). Each mesocosm was filled with tap water one week before the experiment. At day 0 of the experiment, on September 20, 2013, we introduced a different number of carp individuals into the mesocosms: 0 (as a negative control), 3, 4, 5, 7, 9, 11, 17, 25 38, 56, and 85 (in total, 260 individuals).

**b-c.** On days 1, 2, and 3 of the experiment, between 11:00 and 14:00, we collected 15 mL of water in a 50-mL plastic centrifuge tube with a plastic pipette from each mesocosm.

**Experimental procedures**

**7 For each experiment and each experimental group, including controls, provide precise details of all procedures carried out. For example:**

**a. How (e.g., drug formulation and dose, site and route of administration, anaesthesia and analgesia used [including monitoring], surgical procedure, method of euthanasia). Provide details of any specialist equipment used, including supplier(s).**

**b. When (e.g., time of day).**

**c. Where (e.g., home cage, laboratory, water maze).**

**d. Why (e.g., rationale for choice of specific anaesthetic, route of administration, drug dose used).**

We measured the wet-weight of each carp at the end of the experiment (27.6 ± 8.2 g, mean ± SD) and calculated the biomass in each mesocosm. The dead individuals were removed immediately from the mesocosm and frozen as possible as we could. In total, the 11 individuals died in mesocosms with high abundance in the first three days of the experiment, and they were removed immediately.

**Experimental animals**

**8 a. Provide details of the animals used, including species, strain, sex, developmental stage (e.g., mean or median age plus age range), and weight (e.g., mean or median weight plus weight range).**

**b. Provide further relevant information such as the source of animals, international strain nomenclature, genetic modification status (e.g. knock-out or transgenic), genotype, health/immune status, drug- or test naıve, previous procedures, etc.**

Water samples were collected from the mesocosm tank with common carp, *Cyprinus carpio* L., which was purchased from a fish farm.

**Housing and husbandry**

**9 Provide details of:**

**a. Housing (e.g., type of facility, e.g., specific pathogen free (SPF); type of cage or housing; bedding material; number of cage companions; tank shape and material etc. for fish).**

**b. Husbandry conditions (e.g., breeding programme, light/dark cycle, temperature, quality of water etc. for fish, type of food, access to food and water, environmental enrichment).**

**c. Welfare-related assessments and interventions that were carried out before, during, or after the experiment.**

1. Outdoor
2. Mesocosm experiments in twelve concrete outdoor tanks (180 × 100 × 33 cm, volume: 450 L) in the ourdoor condition.
3. Not applicable

**Sample size**

**10 a. Specify the total number of animals used in each experiment and the number of animals in each experimental group.**

**b. Explain how the number of animals was decided. Provide details of any sample size calculation used.**

**c. Indicate the number of independent replications of each experiment, if relevant.**

Not applicable, because we just used water sample for eDNA extraction, not carp individuals.

**Allocating animals to experimental groups**

**11 a. Give full details of how animals were allocated to experimental groups, including randomisation or matching if done.**

**b. Describe the order in which the animals in the different experimental groups were treated and assessed.**

The eDNA was extracted from 15-mL water samples from mesocosms.

**Experimental outcomes**

**12 Clearly define the primary and secondary experimental outcomes assessed (e.g., cell death, molecular markers, behavioural changes).**

Not applicable

**Statistical methods**

**13 a. Provide details of the statistical methods used for each analysis.**

**b. Specify the unit of analysis for each dataset (e.g. single animal, group of animals, single neuron).**

**c. Describe any methods used to assess whether the data met the assumptions of the statistical approach.**

We evaluated the correlation between eDNA concentration and abundance/biomass of carp using a Type II regression with standardized major axis method. Type II regression can treat two variables (x and y) with equal magnitude of random variation. We evaluated the regression for all data collected on the three experimental days, because the trends were very similar between the days and the slopes were not significantly different from each other in our preliminary analysis (p > 0.05). eDNA concentration and carp abundance and biomass were log_10_ transformed to normalize the values based on the results of Shapiro-Wilk normality test (α = 0.05). The R^2^ values of Type II regressions for qPCR and ddPCR were statistically compared using Fisher's z-test. To compare the mean CVs of qPCR and ddPCR, we used Welch t-test. The slopes and intercepts of the Type II regressions between CVs and eDNA concentrations were compared using the 95% confidence intervals between qPCR and ddPCR (α = 0.05). All statistical analyses were set at a significance level of α = 0.05. All statistical analyses and graphics were conducted in R ver. 3.1.0 [26].

**RESULTS**

**Baseline data
14 For each experimental group, report relevant characteristics and health status of animals (e.g., weight, microbiological status, and drug- or test-naıve) before treatment or testing (this information can often be tabulated).**

All animals analyzed were in good health.

**Numbers analysed**

**15 a. Report the number of animals in each group included in each analysis. Report absolute numbers (e.g. 10/20, not 50%).**

100%

**b. If any animals or data were not included in the analysis, explain why.**

Not applicable

**Outcomes and estimation**

**16 Report the results for each analysis carried out, with a measure of precision (e.g., standard error or confidence interval).**

We evaluated the relationship between the eDNA concentrations of common carp and the carp abundance in the mesocosms (Fig. 3). We detected significantly positive correlation between the abundance and DNA copies estimated by qPCR (Type II regression, R^2^ = 0.868, p < 0.0001, y = 0.490*x* – 0.156). The correlation between the carp abundance and DNA copies estimated by ddPCR was significantly positive with larger R^2^ value than qPCR (R^2^ = 0.874, p < 0.0001, y = 0.555*x* – 0.207), but the difference in R^2^ values of qPCR and ddPCR were not statistically significant (Fisher's z test, z = 0.12, p = 0.091).

We also compared the eDNA concentrations and biomass (Fig. 4) and found a significant positive correlation in both qPCR (Type II regression, R^2^ = 0.783, p < 0.0001, y = 0.792*x* + 0.333) and ddPCR (R^2^ = 0.886, p < 0.0001, y = 0.898*x* + 0.251). The R^2^ value was significantly larger in ddPCR than in qPCR (Fisher's z test, z = 2.0, p = 0.040).

**Adverse events**

**17 a. Give details of all important adverse events in each experimental group.**

**b. Describe any modifications to the experimental protocols made to reduce adverse events.**

There were no adverse events.

**DISCUSSION**

**Interpretation/scientific implications**

**18 a. Interpret the results, taking into account the study objectives and hypotheses, current theory, and other relevant studies in the literature.**

**b. Comment on the study limitations including any potential sources of bias, any limitations of the animal model, and the imprecision associated with the results.**

**c. Describe any implications of your experimental methods or findings for the replacement, refinement, or reduction (the 3Rs) of the use of animals in research.**

We found that both qPCR and ddPCR were able to quantify the concentration of carp eDNA along with abundance and biomass gradients (Figs. 3, 4). However, similarly to the previous studies [18, 19, 20, 22], we found lower variation in ddPCR measurements than in qPCR (Fig. 2). The CVs in qPCR were sometimes larger than 50% when estimating eDNA concentration, whereas in ddPCR the variations were about 10% because the ddPCR is able to detect differences as low as 1.25-fold [19]. Thus, the ddPCR is more efficient than qPCR to accurately estimate fish abundance/biomass using eDNA.

**Generalisability/translation**

**19 Comment on whether, and how, the findings of this study are likely to translate to other species or systems, including any relevance to human biology.**

This study only used common carp for the experiment, but the results and discussions can be used for eDNA studies of other organisms.

**Funding**

**20 List all funding sources (including grant number) and the role of the funder(s) in the study.**

This study was supported by the Environment Research and Technology Development Fund (4RF-1302) of the Ministry of the Environment, Japan, JST-CREST, and in part by the Industry-University Collaboration Grant for Young Scientists in Hiroshima University to TT. The funders had no role in study design, data collection and analysis, decision to publish, or preparation of the manuscript.
