# Supplementary material for: Use of Droplet Digital PCR for Estimation of Fish Abundance and Biomass in Environmental DNA Surveys
Source: PLoS One. 2015 Mar 23;10(3):e0122763. doi: 10.1371/journal.pone.0122763 (PMC4370432; doi:10.1371/journal.pone.0122763)
Supplement: S1 Table — Biomass and individuals per mesocosm indicate the final total biomass of the carp in each mesocosm and individuals, which introduced in the mesocosms, respectively. (DOC) [file pone.0122763.s003.doc]

**S1 Table All data used in this study.** Biomass and individuals per mesocosm were measured at the end of the experiment.

| Mesocosm number | Individuals per mesocosm | Biomass per mesocosm (mg) | Experimental day | eDNA copies mL^-1^ (qPCR) | eDNA copies mL^-1^ (ddPCR) |
| --- | --- | --- | --- | --- | --- |
| 1 | 0 | 0 | 1 | 0.16 | 0.00 |
| 2 | 3 | 88.3 | 1 | 106.72 | 54.22 |
| 3 | 4 | 137.2 | 1 | 74.99 | 48.80 |
| 4 | 5 | 90.5 | 1 | 74.51 | 41.71 |
| 5 | 7 | 121.4 | 1 | 313.87 | 139.56 |
| 6 | 9 | 171.2 | 1 | 221.11 | 95.00 |
| 7 | 11 | 197.7 | 1 | 417.54 | 151.67 |
| 8 | 17 | 469.2 | 1 | 170.59 | 77.22 |
| 9 | 25 | 690 | 1 | 301.45 | 113.00 |
| 10 | 38 | 1048.8 | 1 | 871.79 | 303.56 |
| 11 | 56 | 1545.6 | 1 | 1641.14 | 480.00 |
| 12 | 85 | 2346 | 1 | 2827.05 | 755.56 |
| 1 | 0 | 0 | 2 | 0.00 | 0.00 |
| 2 | 3 | 88.3 | 2 | 16.74 | 26.46 |
| 3 | 4 | 137.2 | 2 | 11.07 | 19.24 |
| 4 | 5 | 90.5 | 2 | 3.66 | 14.06 |
| 5 | 7 | 121.4 | 2 | 53.35 | 63.78 |
| 6 | 9 | 171.2 | 2 | 21.77 | 30.34 |
| 7 | 11 | 197.7 | 2 | 79.65 | 77.67 |
| 8 | 17 | 469.2 | 2 | 88.01 | 89.33 |
| 9 | 25 | 690 | 2 | 608.04 | 463.78 |
| 10 | 38 | 1048.8 | 2 | 969.86 | 525.56 |
| 11 | 56 | 1545.6 | 2 | 1081.97 | 628.67 |
| 12 | 85 | 2346 | 2 | 1868.50 | 1084.44 |
| 1 | 0 | 0 | 3 | 0.28 | 0.00 |
| 2 | 3 | 88.3 | 3 | 11.41 | 9.98 |
| 3 | 4 | 137.2 | 3 | 23.48 | 16.75 |
| 4 | 5 | 90.5 | 3 | 6.67 | 6.03 |
| 5 | 7 | 121.4 | 3 | 53.62 | 24.89 |
| 6 | 9 | 171.2 | 3 | 73.81 | 43.00 |
| 7 | 11 | 197.7 | 3 | 86.83 | 40.42 |
| 8 | 17 | 469.2 | 3 | 193.93 | 77.33 |
| 9 | 25 | 690 | 3 | 151.95 | 75.33 |
| 10 | 38 | 1048.8 | 3 | 2449.97 | 1080.00 |
| 11 | 56 | 1545.6 | 3 | 3066.67 | 1224.44 |
| 12 | 85 | 2346 | 3 | 5959.68 | 2047.78 |
