# Supplementary material for: Use of Droplet Digital PCR for Estimation of Fish Abundance and Biomass in Environmental DNA Surveys
Source: PLoS One. 2015 Mar 23;10(3):e0122763. doi: 10.1371/journal.pone.0122763 (PMC4370432; doi:10.1371/journal.pone.0122763)
Supplement: S2 Table — (DOC) [file pone.0122763.s004.doc]

**S2 Table Costs (US$*) for ddPCR and qPCR measurements.**

| Equipment | US$ per 96 well-PCR plate | US$ per 1 well |
| --- | --- | --- |
| **ddPCR (Bio-Rad QX-100)** |  |  |
| 2x ddPCR supermix (Bio-Rad) | 163.334 | 1.701 |
| PCR primers and Taqman probe | 19.056 | 0.198 |
| Droplet cartridge and gasket (Bio-Rad) | 19.847 | 0.207 |
| Droplet Generation Oil (Bio-Rad) | 53.084 | 0.553 |
| Droplet Reader Oil (Bio-Rad) | 196.001 | 2.042 |
| PCR plate | 1.361 | 0.014 |
| PCR-plate foilsheet | 1.191 | 0.012 |
| Total | 451.322 | 4.701 |
|  |  |  |
| **qPCR (Life Technologies, StepOnePlus)** | |  |
| 2x Gene Expression Master Mix | 107.188 | 1.117 |
| PCR primers and Taqman probe | 19.056 | 0.198 |
| PCR plate | 5.104 | 0.053 |
| PCR-plate sheet | 1.191 | 0.012 |
| Total | 132.539 | 1.381 |

*The values were evaluated by Japanese Yen and exchanged for US$ (1 Japanese Yen = 0.008507 US$, on 27 Nov. 2014).
