# Supplementary material for: Use of Droplet Digital PCR for Estimation of Fish Abundance and Biomass in Environmental DNA Surveys
Source: PLoS One. 2015 Mar 23;10(3):e0122763. doi: 10.1371/journal.pone.0122763 (PMC4370432; doi:10.1371/journal.pone.0122763)
Supplement: S3 Table — (DOC) [file pone.0122763.s005.doc]

**S3 Table Estimated time for ddPCR and qPCR measurements**

| Measurement steps | Time |
| --- | --- |
| **ddPCR (Bio-Rad QX-100) for 48 wells for unknown samples** | |
| PCR sample and droplet preparation by Droplet generator | 1:10 |
| PCR (40 cycles, ramp rate: 2.5°C s^-1^) by GeneAmp 9700 | 3:10 |
| Droplet florescence reading by Droplet reader | 1:20 |
| Total | 5:40 |
|  |  |
| **qPCR (Life Technologies, StepOnePlus) for 81 wells for unknown samples with standards (n=15)** | |
| PCR sample preparation | 0:40 |
| PCR (40 cycles, ramp rate: 5°C s^-1^) and florescence reading | 1:40 |
| Total | 2:20 |
